# A Novel Blue Light Laser (445 nm) in Non-surgical Treatment of Chronic Periodontitis: A Clinical and Microbiological Study

**DOI:** 10.7759/cureus.67252

**Published:** 2024-08-19

**Authors:** Indira Mujić Jahić, Mirjana Gojkov Vukelić, Sanja Hadžić, Enes Pašić, Arma Muharemović, Lana Salihefendić, Rijad Konjhodžić

**Affiliations:** 1 Department of Periodontology and Oral Medicine, Faculty of Dentistry with the Dental-Clinical Center, University of Sarajevo, Sarajevo, BIH; 2 Alea Genetic Center, Health Institute Alea Dr. Kandić, Sarajevo, BIH

**Keywords:** scaling and root planing, clinical and microbiological study, laser, dental surgery, chronic periodontitis

## Abstract

Objective: This comprehensive research aimed to thoroughly examine the effectiveness of a diode laser (445 nm) in combination with non-surgical treatment in patients with chronic periodontitis (CP) by evaluating a wide range of clinical and microbiological parameters.

Materials and methods: Thirty-one subjects diagnosed with CP were included in this study. The total number of treated periodontal pockets was 862. The subjects were randomly assigned to group 1, which underwent scaling and root planing and laser therapy (SRP+L), and group 2, which underwent scaling and root planing (SRP) only. All respondents underwent a periodontal diagnostic protocol. The parameters plaque index (PI), gingival index (GI), bleeding on probing index (BOP), probing depth (PD), clinical attachment level (CAL), and tooth mobility (TM) were registered. Clinical periodontal measurements were performed at baseline and one and three months after therapy. Microbiological analysis was conducted on *Aggregatibacter actinomycetemcomitans* (Aa), *Porphyromonas gingivalis* (Pg), and *Tannerella forsythia* (Tf) using real-time polymerase chain reaction (PCR). For microbiological analysis, samples were taken at baseline, immediately after therapy, and after three months. Laser irradiation was performed immediately after SRP.

Results: All clinical parameters improved statistically from baseline to three months after therapy. For all examined clinical parameters, better results were achieved in group 1 than in group 2. This study showed a more significant reduction in Pg and Tf from baseline to three months in group 1 compared to group 2.

Conclusion: These results showed that the diode laser wavelength 445 nm was also usable in treating periodontal diseases as an additional method to SRP.

## Introduction

Chronic periodontitis (CP) is the most common form of periodontal disease, caused by specific periodontopathogenic bacteria and their virulence factors [1-3]. *Aggregatibacter actinomycetemcomitans* (Aa) is the primary causative agent of aggressive periodontitis (AP), while *Porphyromonas gingivalis* (Pg) and *Tannerella forsythia* (Tf) are also implicated in CP [4,5].

Microbiological analysis and clinical parameters are essential for diagnosing and monitoring periodontitis therapy [4]. Polymerase chain reaction (PCR) is a highly sensitive technique considered the "gold standard" for detecting and quantifying periodontal pathogens, providing crucial data for evaluating therapeutic success [5].

Standard non-surgical periodontal treatment, including scaling and root planing (SRP), is the basic protocol for CP patients [6,7]. However, SRP alone does not ensure the complete elimination of periodontal pathogens, as their toxins can penetrate the periodontal pockets and cause disease recurrence [8,9].

Lasers have been shown to enhance periodontal treatment and are used in other areas of dentistry. Research into laser use in dentistry began in the 1960s and has intensified since the 1990s [10]. Lasers have demonstrated antimicrobial, anti-inflammatory, analgesic, and anti-edematous effects on periodontal tissue, improving the clinical picture of periodontal diseases and reducing pathogenic microorganisms [3,4,11].

While several studies have demonstrated the superiority of diode laser therapy over SRP alone for CP, some authors view diode lasers as merely supportive in non-surgical or surgical periodontal therapy [3,11-13]. New lasers with different wavelengths, including a 445 nm dental diode laser, are now available, offering opportunities to study their effectiveness compared to previous wavelengths. This laser operates at three wavelengths: 970 nm, 660 nm, and a blue beam at 445 nm [14].

This research aimed to evaluate the effectiveness of a 445 nm diode laser in combination with non-surgical treatment for CP by assessing clinical and microbiological parameters using real-time PCR for three periodontal pathogens: Aa, Pg, and Tf.

## Materials and methods

The present study was a prospective, single-center study conducted from February to December 2020 at the Institute/Clinic for Oral Medicine and Periodontology, Faculty of Dentistry with the Dental-Clinical Center in Sarajevo, Bosnia and Herzegovina. The research was approved by an Institutional Ethical Review Board (approval number: 02-3-4-59-1-5/2020) on February 17, 2020. All participants signed informed consent for voluntary participation. The sample size was determined using G*Power, estimating an effect size of 0.50 with 85% power and a 0.05 alpha level (a priori analysis) to ensure compatibility with other studies [11-13].

The study included 31 subjects, aged 35-65, diagnosed with chronic periodontitis (CP) and having at least two probing pockets with a depth (PD) ≥ 5 mm. In total, 451 teeth were treated, encompassing 862 periodontal pockets (mesial and distal sides of each tooth with PD > 4 mm).

Inclusion criteria were as follows: first-time CP patients seeking treatment, no periodontal intervention in the past year, aged 35-65, having at least two periodontal pockets ≥ 5 mm, and being generally healthy without regular therapy.

Exclusion criteria were as follows: periodontal therapy within the past year, antibiotic therapy within the past six months, systemic illnesses, pregnancy, and smoking.

Study protocol

The subjects were randomly assigned to two groups: group 1 underwent SRP and laser therapy (SRP+L) (diode laser 445 nm), and group 2 underwent SRP only, with an approximately equal number of treated periodontal pockets in each group. Before therapy, all participants underwent a periodontal anamnestic diagnostic protocol and clinical radiological evaluation. Initial treatment, including oral hygiene instructions and guidance for taking panoramic X-rays, was performed during the first visit. During the second visit, oral hygiene was evaluated, and the panoramic X-ray image was analyzed. In the third visit, samples were taken for microbiological analysis, and therapy was administered based on the patient's group classification (SRP or SRP+L).

In group 1, scaling and root planing were performed using a diode laser with a blue laser beam of 445 nm wavelength, the SIROLaser Blue (Dentsply Sirona, Bensheim, Germany). Each tooth's mesial and distal sides with PD > 4 mm were treated. Laser irradiation was performed immediately after SRP. Irradiation of the periodontal pocket was performed with EasyTip 320µm (Dentsply Sirona). The laser tip was moved manually, following the manufacturer's instructions: always in a sigmoid motion from the bottom of the pocket (non-contact) upward. One movement within the periodontal pocket lasted 4-5 seconds. The movements were repeated five times, with a five-second pause between repetitions. The total active laser time per pocket was 20-25 seconds.

The spot size and beam characteristics are according to the manufacturer's instructions.

The parameters for the laser were set as follows: 445 nm, 20-50 Hz, duty cycle 50% (pulse mode), average power 1-1.5 W, and using a 320-micron tip for periodontal pocket irradiation.

Following the recommended precautionary measures during work with a laser, the patient, the therapist, and the dental nurse wore protective glasses, and the area where the intervention was performed was appropriately marked.

Clinical parameter assessment

Clinical periodontal measurements were performed at baseline and one and three months post-therapy. The standard parameters evaluated included plaque index (PI), gingival index (GI), bleeding on probing (BOP), and probing depth (PD). Clinical attachment level (CAL) and tooth mobility (TM) were measured only at baseline and at three months post-therapy. The Williams probe was used for these assessments. All clinical parameters and results were recorded in patient records designed for this research.

Microbiological analysis of periodontal pathogens

Microbiological analysis focused on *Aggregatibacter actinomycetemcomitans* (Aa), *Porphyromonas gingivalis* (Pg), and *Tannerella forsythia* (Tf) using real-time PCR, a leading method in microbiology. Samples were collected at baseline, immediately post-therapy, and three months post-therapy. Sample sites were isolated with cotton rolls, dried, and sampled using sterile paper points placed in periodontal pockets for 10 seconds, avoiding contact with saliva or mucous membranes. The samples were then transported in Eppendorf tubes to a specialized laboratory.

All samples were analyzed using the SYBR Green method at the Alea Genetic Center, Sarajevo. DNA extraction was performed with Lucigen QuickExtract™ DNA Extraction Solution (LGC Biosearch Technologies, Hoddesdon, UK) and quantified using a DNA high-sensitivity kit on a Qubit Fluorometer 3 (ThermoFisher Scientific, Waltham, MA). Detection was carried out using the KAPA SYBR® FAST qPCR Master Mix (2X) Kit (Kapa Biosystems, Wilmington, MA) on a Bio-Rad CFX96 instrument (Bio-Rad, Hercules, CA). Data analysis was conducted with CFX Manager Software, comparing bacterial nucleic acid levels. A cutoff value of 36 was used to determine the presence of bacteria, with values above 36 considered unfavorable or below detection levels.

Statistical analysis

The results were analyzed using standard statistical methods with the Statistical Package for the Social Sciences (SPSS) version 21.0 (IBM SPSS Statistics, Armonk, NY). Data are presented as mean values (X) and standard deviations (SD), as well as medians and interquartile ranges (25th-75th percentiles). The Kolmogorov-Smirnov test assessed the deviation from normal distribution.

Comparative analysis for independent numerical variables was conducted using Student's t-test for variables that met normal distribution conditions or the non-parametric Mann-Whitney U test for variables with irregular distribution. Dependent numerical variables were analyzed using one-way repeated measures analysis of variance (ANOVA), paired t-test, Friedman test, and Wilcoxon test. A p-value of <0.05 was considered statistically significant.

## Results

Table 1 shows the baseline characteristics for the examined groups. Before treatment, group 1 and group 2 showed significant differences across several clinical parameters. Group 1 had a higher PI with a mean value of 1.85±0.03 compared to 1.39±0.04 in group 2 (p=0.000). The GI was also higher in group 1, averaging 1.97±0.02 versus 1.60±0.03 in group 2 (p=0.000). BOP was more pronounced in group 1 with values of 2.45±0.03 compared to 2.15±0.04 in group 2 (p=0.000). PPD and CAL were also significantly different. Group 1 had a median PPD of 4.0 (range: 4.0-5.0), while group 2 had 3.0 (range: 4.0-5.0) (p=0.013). CAL was 3.0 (range: 3.0-4.0) in group 1 compared to 2.0 (range: 2.0-3.25) in group 2 (p=0.001). Additionally, TM was higher in group 1 with a mean value of 0.27±0.03 versus 0.10±0.02 in group 2 (p=0.001).

**Table 1:** Baseline value of clinical parameters between examined groups Data expressed as mean (±SD) and median (IQR) PI: plaque Index, GI: gingival index, BOP: bleeding on probing, PD: probing depth, CAL: clinical attachment level, TM: tooth mobility, SD: standard deviation, IQR: interquartile range *p<0.05, **p<0.001

Parameter	Before treatment		
	Group 1	Group 2	p-value
PI	1.85±0.03	1.39±0.04	0.000**
GI	1.97±0.02	1.60±0.03	0.000**
BOP	2.45±0.03	2.15±0.04	0.000**
PPD	4.0 (4.0-5.0)	3.0 (4.0-5.0)	0.013*
CAL	3.0 (3.0-4.0)	2.0 (2.0-3.25)	0.001**
TM	0.27±0.03	0.10±0.02	0.001**

In group 1, the PI value decreased from 1.85±0.03 before treatment to 0.34±0.003 one month after (p=0.000) and to 0.45±0.003 at three months (p=0.000 versus pre-treatment, p=0.018 versus one month). The GI value decreased from 1.97±0.02 to 0.95±0.03 at one month (p=0.000) and 0.68±0.02 at three months (p=0.000 for both). The BOP value dropped from 2.45±0.03 to 1.0±0.04 at one month (p=0.000) and to 0.81±0.03 at three months (p=0.000 for both). The PPD value decreased from 4.61±0.06 to 2.82±0.05 at one month (p=0.000) and 2.71±0.03 at three months (p=0.041). The CAL value was 3.0 (3.0-4.0) before treatment and 3.0 (3.0-4.0) at three months (p=0.886). The TM value decreased from 0.27±0.03 before treatment to 0.14±0.02 at three months (p=0.000) (Table 2).

**Table 2:** Trend of changes in the clinical parameters in group 1 Data expressed as mean (±SD) and median (IQR) PI: plaque index, GI: gingival index, BOP: bleeding on probing, PD: probing depth, CAL: clinical attachment level, TM: tooth mobility, SD: standard deviation, IQR: interquartile range *Compared to before treatment, ^#^compared to one month after treatment

Parameter	Before treatment	One month after treatment	p-value	Three months after treatment	p-value
PI	1.85±0.03	0.34±0.003	*0.000	0.45±0.003	*0.000
					^#^0.018
GI	1.97±0.02	0.95±0.03	*0.000	0.68±0.02	*0.000
					^#^0.000
BOP	2.45±0.03	1.0±0.04	*0.000	0.81±0.03	*0.000
					^#^0.000
PD	4.61±0.06	2.82±0.05	*0.000	2.71±0.03	*0.000
					^#^0.041
CAL	3.0 (3.0-4.0)	-	-	3.0 (3.0-4.0)	*0.886
TM	0.27±0.03	-	-	0.14±0.02	*0.000

In group 2, the PI value decreased from 1.39±0.04 before treatment to 0.76±0.003 one month after (p=0.000) and to 0.87±0.003 at three months (p=0.000 versus pre-treatment, p=0.000 versus one month). The GI value decreased from 1.60±0.03 to 1.29±0.02 at one month (p=0.000) and 1.21±0.02 at three months (p=0.000 versus pre-treatment, p=0.036 versus one month). The BOP value dropped from 2.15±0.04 to 1.23±0.04 at one month (p=0.000) and 1.31±0.03 at three months (p=0.000 versus pre-treatment, p=0.044 versus one month). The PD value decreased from 4.40±0.05 to 3.53±0.05 at one month (p=0.000) and to 3.48±0.03 at three months (p=0.000 versus pre-treatment, p=0.094 versus one month). The CAL value was 2.0 (2.0-3.25) before treatment and 2.5 (2.0-3.25) at three months (p=0.108). The TM value decreased from 0.10±0.02 before treatment to 0.06±0.01 at three months (p=0.000) (Table 3).

**Table 3:** Trend of changes in the clinical parameters in group 2 Data expressed as mean (±SD) and median (IQR) PI: plaque index, GI: gingival index, BOP: bleeding on probing, PD: probing depth, CAL: clinical attachment level, TM: tooth mobility, SD: standard deviation, IQR: interquartile range *Compared to before treatment, ^#^compared to one month after treatment

Parameter	Before treatment	One month after treatment	p-value	Three months after treatment	p-value
PI	1.39±0.04	0.76±0.003	*0.000	0.87±0.003	*0.000
					^#^0.000
GI	1.60±0.03	1.29±0.02	*0.000	1.21±0.02	*0.000
					^#^0.000
BOP	2.15±0.04	1.23±0.04	*0.000	1.31±0.03	*0.000
					^#^0.044
PD	4.40±0.05	3.53±0.05	*0.000	3.48±0.03	*0.000
					^#^0.094
CAL	2.0 (2.0-3.25)	-	-	2.5 (2.0-3.25)	*0.108
TM	0.10±0.02	-	-	0.06±0.01	*0.000

Subgingival plaque samples were collected at baseline, immediately after therapy, and three months postoperatively for microbiological analysis. In group 1, Aa values were 35.42±0.20 before treatment, 35.91±0.08 immediately after, and 35.81±0.49 after three months, showing no significant change (p=0.368). Pg increased from 21.26 to 27.07 immediately after treatment (p=0.008) and to 31.25 after three months (p=0.012) but was not significantly different from the immediate post-treatment value (p=0.093). Tf increased from 21.58±1.54 to 26.22±1.55 immediately after treatment (p=0.002) and remained high after three months (26.30±1.57, p=0.003), with no significant change between immediate and three months post-treatment (p=0.638) (Table 4).

**Table 4:** Trend of changes in Aa, Pg, and Tf in the examined groups in the monitoring period Data expressed as mean (±SD) and median (IQR) Aa: *Aggregatibacter actinomycetemcomitans*, Pg: *Porphyromonas gingivalis*, Tf: *Tannerella forsythia*, SD: standard deviation, IQR: interquartile range *Compared to before treatment, ^#^compared to immediately after treatment

	Before treatment	Immediately after treatment	p-value	Three months after treatment	p-value
Group 1					
Aa	35.42±0.20	35.91±0.08	*0.368	35.81±0.49	*0.368
					^#^0.368
Pg	21.26 (16.81-24.86)	27.07 (25.83-32.90)	*0.008	31.25 (27.87-34.88)	*0.012
					^#^0.093
Tf	21.58±1.54	26.22±1.55	*0.002	26.30±1.57	*0.003
					^#^0.638
Group 2					
Aa	25.40±0.84	29.97±2.17	*0.06	28.56±2.90	*0.06
					^#^0.06
Pg	23.03 (17.61-31.76)	28.70 (23.73-31.68)	*0.345	24.22 (16.12-35.04)	*0.345
					^#^0.345
Tf	23.31±1.68	27.90±1.03	*0.023	23.64±2.13	*0.583
					^#^0.071

In group 2, Aa values were 25.40±0.84 before treatment, 29.97±2.17 immediately after, and 28.56±2.90 after three months, with no significant change (p=0.06). Pg values changed from 23.03 to 28.70 immediately after treatment and to 24.22 after three months, showing no significant trend. Tf increased from 23.31±1.68 to 27.90±1.03 immediately after treatment (p=0.023) and to 23.64±2.13 after three months, with no significant differences from baseline (p=0.583) or immediate post-treatment values (p=0.071) (Table 4).

## Discussion

The SIROLaser Blue, a new diode laser, uniquely employs blue light at 445 nm alongside the conventional infrared and red light (600-970 nm). This laser, featuring three diodes at 445, 660, and 970 nm, offers several advantages, including higher absorption in tissue pigments, focused energy application, reduced energy dissipation, and minimized collateral damage. Despite these potential benefits, few studies have explored the effectiveness of blue light in periodontology [3,4].

Previous studies indicate that laser treatment combined with SRP enhances clinical, microbiological, and immunohistochemical outcomes in periodontal disease treatment, promoting faster healing [2,3,8,9,15-17]. This study evaluated the effectiveness of the 445 nm diode laser in conjunction with non-surgical treatment for chronic periodontitis by assessing clinical and microbiological parameters.

Our study demonstrated significant improvements in all clinical parameters from baseline to three months in both groups, consistent with prior research using diode lasers of different wavelengths (660-980 nm) [3,12,16-20]. While some studies used blue wavelengths for periodontal pocket treatment, they primarily utilized in vitro models [21,22]. Kulińska-Michalska et al. [23] confirmed the effectiveness of periodontal treatment by combining SRP with photodynamic therapy or SRP with laser irradiation, compared to SRP alone, which corresponds to the results of this research and those of other authors [3,15,17,24]. Similarly, Pawelczyk-Madalińska et al. [8] concluded that diode lasers (808-980 nm) enhance SRP outcomes, corroborating our results.

Our findings showed a significant reduction in PD in group 1 compared to group 2. This is consistent with the results of Kaur et al. [25], who found significant improvements in clinical parameters using an 810 nm diode laser in addition to SRP. These results suggest that combining a diode laser with SRP yields better clinical outcomes, aligning with our research and other studies [26,27].

The results from our research align with previous studies, demonstrating that the 445 nm blue laser is particularly effective in treating periodontal diseases. This effectiveness stems from its superior tissue absorption, potent antimicrobial properties, reduced thermal damage, and enhanced precision during procedures. Furthermore, the 445 nm blue laser cuts soft tissue more rapidly and efficiently, with less energy dispersion than the commonly used wavelengths of 800-980 nm [21].

Bacterial infection triggers the chronic inflammatory response in periodontal tissues; thus, eliminating periodontal pathogens is crucial for reducing PD. Numerous studies have demonstrated the antimicrobial effects of diode lasers on periodontal pathogens [3,17,20]. In our study, Aa was barely detectable, while Pg and Tf were more prevalent, consistent with their roles in CP [4,5].

In group 1, Pg values significantly increased immediately after treatment and remained elevated after three months. In group 2, Pg values rose immediately post-treatment but returned to baseline levels after three months, showing no significant trend. Tf values also increased significantly immediately and three months post-treatment compared to baseline. These findings suggest that the 445 nm blue laser effectively reduces Pg and Tf and can be a beneficial adjunct to SRP in CP treatment, aligning with other studies.

Mallineni et al. [28] reported similar findings, demonstrating significant differences in Pg levels when comparing SRP alone to SRP with photodynamic laser therapy. Kamma et al. [29] found that diode laser treatment combined with SRP was more effective than SRP or laser alone in reducing microbial and clinical parameters in aggressive periodontitis. Grzech-Leśniak et al. [19] also observed similar clinical and microbiological improvements using the erbium (Er) laser compared to SRP and photodynamic therapy (635 nm).

Although this study demonstrates a positive effect of using the 445 nm blue light laser with standard non-surgical protocols in treating chronic periodontitis, several limitations should be noted. First, the three-month study duration may be insufficient to evaluate the long-term effects on periodontal stability and relapse prevention. Second, the relatively small and homogeneous sample size could affect the external validity of the findings.

Future studies should consider a longer follow-up period than three months to evaluate the long-term effects on periodontal stability and prevention of relapse. Additionally, more research is needed to clarify the impact of blue laser light in various therapeutic approaches for treating periodontal diseases. Future research topics could include the impact of blue laser light in combination with regenerative treatment methods in non-surgical protocols and periodontal surgery.

## Conclusions

This study shows that using a 445 nm blue diode laser with standard scaling and root planing (SRP) significantly improves clinical parameters and reduces periodontal pathogens compared to SRP alone. The 445 nm blue diode laser is an effective adjunctive treatment for periodontal diseases, enhancing non-surgical therapy outcomes.

Given that most prior studies on the 445 nm diode laser were ex vivo or in vitro, our in vivo evidence is significant. Further research with longer follow-ups and larger sample sizes is needed to confirm these findings and explore long-term benefits.
